# The NuA4 acetyltransferase and histone H4 acetylation promote replication recovery after topoisomerase I-poisoning

**DOI:** 10.1186/s13072-019-0271-z

**Published:** 2019-04-16

**Authors:** Chiaki Noguchi, Tanu Singh, Melissa A. Ziegler, Jasmine D. Peake, Lyne Khair, Ana Aza, Toru M. Nakamura, Eishi Noguchi

**Affiliations:** 1 Department of Biochemistry and Molecular Biology, Drexel University College of Medicine, 245 N. 15th Street, Philadelphia, PA 19102 USA; 2 Department of Biochemistry and Molecular Genetics, University of Illinois at Chicago, Chicago, IL 60607 USA; 3 Present Address: Fox Chase Cancer Center, Philadelphia, USA; 4 Present Address: University of Massachusetts Medical School, Worcester, USA

**Keywords:** Histone H4, Acetylation, DNA replication, Replication forks, Chromatin, *Schizosaccharomyces pombe*, NuA4, Vid21, Mst1, Swi1, Timeless, Replication fork protection complex

## Abstract

**Background:**

Histone acetylation plays an important role in DNA replication and repair because replicating chromatin is subject to dynamic changes in its structures. However, its precise mechanism remains elusive. In this report, we describe roles of the NuA4 acetyltransferase and histone H4 acetylation in replication fork protection in the fission yeast *Schizosaccharomyces pombe*.

**Results:**

Downregulation of NuA4 subunits renders cells highly sensitive to camptothecin, a compound that induces replication fork breakage. Defects in NuA4 function or mutations in histone H4 acetylation sites lead to impaired recovery of collapsed replication forks and elevated levels of Rad52 DNA repair foci, indicating the role of histone H4 acetylation in DNA replication and fork repair. We also show that Vid21 interacts with the Swi1–Swi3 replication fork protection complex and that Swi1 stabilizes Vid21 and promotes efficient histone H4 acetylation. Furthermore, our genetic analysis demonstrates that loss of Swi1 further sensitizes NuA4 and histone H4 mutant cells to replication fork breakage.

**Conclusion:**

Considering that Swi1 plays a critical role in replication fork protection, our results indicate that NuA4 and histone H4 acetylation promote repair of broken DNA replication forks.

**Electronic supplementary material:**

The online version of this article (10.1186/s13072-019-0271-z) contains supplementary material, which is available to authorized users.

## Background

Chromatin structure plays essential roles in most DNA transactions including transcription, DNA replication, repair, and recombination. The dynamic changes in chromatin structure are regulated by post-translational modifications of histones. It is well understood that histone modifications modulate gene expression [1]. However, mechanisms by which histone modifications regulate DNA replication and DNA damage response still remain elusive [2].

Previous studies demonstrated that histone acetylation plays critical roles in DNA replication and repair. In *S. cerevisiae*, histone H3 and H4 acetylation is dynamically regulated around an origin of replication [3]. *S. cerevisiae* cells harboring mutations simultaneously at five acetylation sites (K9R/K14R of H3 and K5R/K8R/K12R of H4) have defects in S-phase progression and efficient origin firing, indicating that acetylation of multiple lysine residues of H3 and H4 tails plays an important role in DNA replication [3]. In addition to N-terminal tail acetylation, H3 is acetylated at lysine 56 (H3K56), which promotes chromatin assembly during S-phase [4] and restores chromatin structure following DNA double-strand break (DSB) repair [5]. H4 acetylation is also involved in genome maintenance. The TIP60 histone acetyltransferase (HAT) complex and its yeast ortholog NuA4 complex, both of which are responsible for histone H4 acetylation, play an important role in DNA repair in conjunction with other histone modifications that serve as signals to recruit these proteins. NuA4 acetylates nucleosomal H4 at four lysine residues at position 5, 8, 12 and 16 [2]. NuA4 is also involved in acetylation of H2A and the H2A variant H2AZ [6–9]. Moreover, the TIP60 complex is required for the incorporation of H2AZ into the nucleosome, which is important in DNA damage response [10, 11]. Earlier studies demonstrated that DSB-dependent phosphorylation of H2A (H2AX in higher eukaryotes) serine 129 results in recruitment of chromatin-remodeling complexes including NuA4 and that phosphorylation of H4 serine 1 inhibits NuA4 association [12–14]. More recently, a study reported that NuA4 subunits are recruited even in the absence of H2A phosphorylation in G1- and G2/M-arrested cells and that, rather, reduced H2A phosphorylation enhanced recruitment of NuA4 subunits [15]. Therefore, although the role of NuA4 in DNA replication and repair is still elusive, these studies suggest that coordinating NuA4 association and dissociation at DSB is involved in setting up local chromatin conformational change to promote DNA repair processes.

NuA4 consists of a number of subunits including Esa1 (the catalytic subunit), and Vid21/Eaf1 (a regulatory subunit) [16]. In *S. cerevisiae*, Vid21 acts as a platform to assemble NuA4 subunits, facilitating histone acetylation [17, 18]. Mutations in Esa1 or Vid21 render cells highly sensitive to DNA-damaging agents [17, 19–21]. Esa1 is required for DSB repair via homologous recombination and non-homologous end-joining pathways [19]. The mammalian Esa1 ortholog Tip60 is also required for DSB repair [10, 11, 22, 23], indicating that the roles of NuA4-dependent histone acetylation in DNA damage response are conserved among species. *S. pombe* also contains homologs of NuA4 components including Mst1 (Esa1 ortholog) and Vid21 (Vid21/Eaf1 ortholog), although whether these proteins form a complex is still elusive [24, 25]. *mst1* temperature-sensitive mutant cells show significant sensitivity to various DNA-damaging agents and accumulate Rad52 DNA damage foci [25], suggesting the role of NuA4 in DNA damage response in *S. pombe*. NuA4-targeted acetylation sites appeared to also be important for DNA damage response in *S. pombe*. Studies have shown that mutations in H4 simultaneously at NuA4-targeted sites K8 and K16 render cells sensitive to hydroxyurea, a DNA replication inhibitor [26]; however, the role of NuA4 and histone H4 acetylation in DNA replication and repair is largely unknown in this organism.

Recent studies started delving into the role of NuA4 in DNA replication and repair in *S. cerevisiae*. Mutations in NuA4 subunits are epistatic to a mutation in *rev3*, the gene encoding the error-prone DNA polymerase ζ, for sensitivity to the alkylating agent methyl methanesulfonate (MMS) and replication recovery from MMS treatment [27]. In addition, mutations in H4 acetylation sites, H4 HATs (including NuA4), or H4 histone deacetylases (HDACs) cause increased levels of trinucleotide repeat expansions, which is suppressed by mutations in RAD5, RAD52, and RAD57. On the other hand, mutations in NuA4 or H4 acetylation sites suppress sister chromatid recombination [28]. These genetic results suggest that histone H4 regulation is important in maintaining trinucleotide repeat stability and promoting sister chromatid recombination probably via post-replication repair events. However, the molecular mechanisms by which H4 acetylation and NuA4 regulate DNA replication and repair are still poorly understood.

In this study, we report that mutations in NuA4 subunits and histone H4 acetylation sites render cells highly sensitive to camptothecin (CPT), a topoisomerase poison that causes replication fork breakage [29, 30]. These mutants show defects in recovery of collapsed replication forks. We also show that Vid21 interacts with the Swi1–Swi3 replication fork protection complex (FPC), which plays a critical role in stabilization of replication fork and efficient activation of the replication checkpoint in response to replication stress [31, 32]. Loss of Swi1 leads to unstable Vid21, leading to a reduction in histone H4 acetylation. These results indicate important roles of NuA4 and histone H4 acetylation in DNA replication and repair processes.

## Results

### NuA4 is required for cellular tolerance to camptothecin-induced replication fork breakage in *S. pombe*

We previously reported the involvement of Vid21 and Mst1 in S-phase stress response in *S. pombe*. Although these proteins are homologs of *S. cerevisiae* NuA4 subunits Vid21/Eaf1 and Esa1, respectively, whether they interact together is unknown [25, 33]. Therefore, we first confirmed the interaction of these proteins via immunoprecipitation. Accordingly, *S. pombe* cells were engineered to express Vid21-13Myc and Mst1-5FLAG from their native promoters. There was no obvious growth defect in these cells. Considering that Vid21 and Mst1 are essential for *S. pombe* growth [34], we concluded that Vid21-13Myc and Mst1-5FLAG are functional. Mst1-5FLAG efficiently coprecipitated with Vid21-13Myc from *S. pombe* cell extracts (Additional file 1: Supplementary Fig. S1), indicating that Vid21 interacts with Mst1 and that these proteins are part of the NuA4 complex in *S. pombe* cells.

**Fig. 1:**
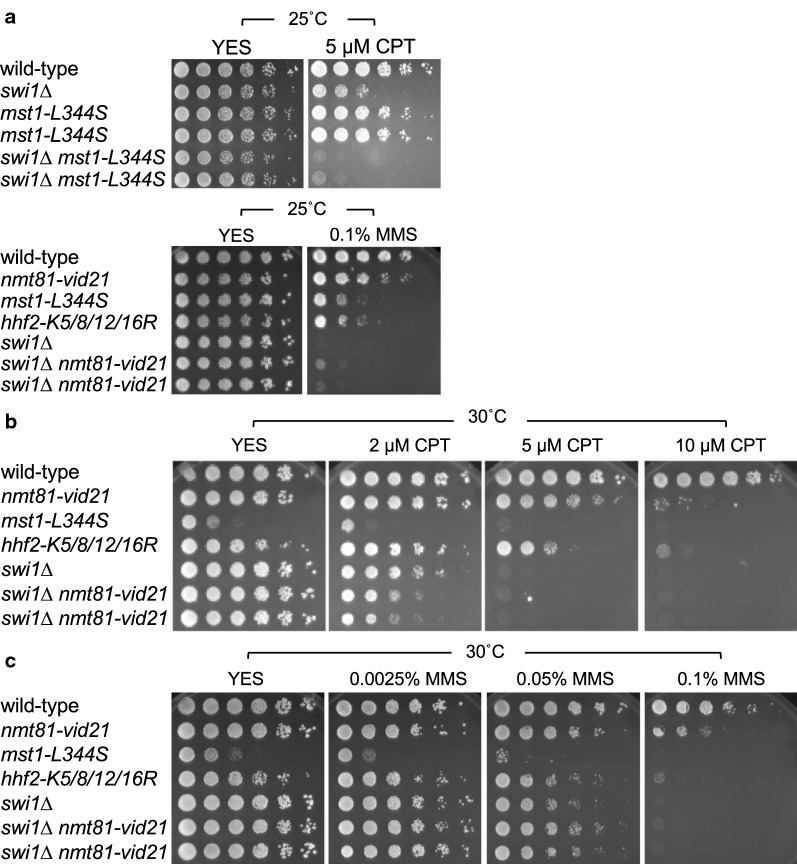
NuA4 is required for cellular tolerance to camptothecin (CPT). **a**–**c** Fivefold dilutions of cells with the indicated genotypes were incubated on YES agar medium supplemented with the indicated concentration of CPT or MMS for 3–5 days at the indicated temperature. Representative images of repeat experiments are shown

To investigate the role of NuA4 in DNA replication and repair in *S. pombe*, we evaluated whether NuA4 mutants are sensitive to camptothecin (CPT), a topoisomerase I poison that causes replication fork breakage. A previous study reported that temperature-sensitive *mst1-L344S* cells were not significantly sensitive to CPT; however, in this previous study, *mst1-L344S* was expressed from the *nmt1*^+^ promoter integrated at the *leu1*^+^ locus [25]. Therefore, in order to conduct experiments in a more physiological condition, in our current study, *mst1-L344S* was expressed from its native promoter integrated at its own genetic locus [24]. We first tested CPT sensitivity of *mst1-L344S* mutant cells at 25 °C, which does not hamper growth of these cells (Fig. 1a). At this temperature, *mst1-L344S* cells were not significantly sensitive to CPT. We next incubated cells at 30 °C, a temperature normally used to grow *S. pombe* cells (Fig. 1b). Wild-type cells showed no obvious growth defects in the presence of up to 10 µM CPT. *mst1-L344S* cells lost fitness at 30 °C due to the temperature sensitivity, although these cells were still able to grow in the absence of CPT. However, *mst1-L344S* cells failed to grow in the presence of 5 µM CPT (Fig. 1b). We then determined the effect of *swi1* deletion on cellular tolerance to CPT because Swi1 plays a central role in replication fork stabilization [31, 32]. As previously reported, *swi1*∆ cells are sensitive to CPT due to defects in replication fork protection (Fig. 1a). Interestingly, *swi1*∆ *mst1-L344S* double mutant cells were much more sensitive to 5 µM CPT than either single mutant at 25 °C, where *mst1-L344S* cells have no obvious growth defect under this condition (Fig. 1a), suggesting the involvement of NuA4 in replication fork protection.

Next, the *vid21* promoter was replaced with the thiamine-repressible *nmt81* promoter to control the level of Vid21 expression (*nmt81-vid21*) [24]. The cellular amount of Vid21 was greatly reduced when *nmt81-vid21* cells were grown in the presence of thiamine [24]. Therefore, all experiments were performed using regular YES growth medium that contains thiamine. As shown in Fig. 1b, *nmt81-vid21* cells were sensitive to CPT. We then explored the genetic interactions between *swi1*∆ and *nmt81-vid21* in cellular tolerance to replication fork breakage caused by CPT. Again, *swi1*∆ *nmt81-vid21* double mutant cells were much more sensitive to CPT than either single mutant (Fig. 1b). These results are consistent with the notion that NuA4 components Mst1 and Vid21 play an important role in repair of collapsed replication forks induced by CPT.

CPT traps topoisomerase I on DNA and induce replication fork breakage [35]. In order to address the role of topoisomerase I (Top1 in *S. pombe*) in the campothecin sensitivity of NuA4 mutants, we introduced *top1*∆ into *nmt81-vid21* and *mst1-L344S* cells. Importantly, *top1*∆ suppressed CPT sensitivity of *nmt81-vid21* and *mst1-L344S* cells (Additional file 1: Supplementary Fig. S2). Thus, our findings indicate that Swi1 and NuA4 play an important role in protection and/or repair of broken replication forks induced by Top1-DNA covalent complexes.

### Roles of NuA4 in response to fork-stalling agents

The aforementioned results are in striking contrast to our previous results where we used hydroxyurea (HU) to understand the role of histone H4 acetylation in DNA replication [24]. HU slows down replication and has different effects than those of CPT, which induces fork breakage. We showed that *mst1-L344S* and *nmt81-vid21* cells were not significantly sensitive to HU. Interestingly, unlike the case for CPT sensitivity, *mst1-L344S* and *nmt81-vid21* mutations partially suppressed HU sensitivity of *swi1*∆ cells [24]. Thus, depending on the types of replication stress, NuA4 may use different mechanisms to regulate replication fork integrity.

To further understand the mechanism by which NuA4 regulates DNA damage response during DNA replication, we performed MMS sensitivity assays. The alkylating agent MMS is believed to slow down replication fork progression [36–38]. Interestingly, *mst1-L344S* and *nmt81-vid21* cells were sensitive to MMS (Fig. 1a). However, unlike the case of CPT, there was no additive MMS sensitivity between *swi1*∆ and *nmt81-vid21* (Fig. 1a, c). Rather, similar to the effect of HU [24], *nmt81-vid21* partially rescued the MMS sensitivity of *swi1*∆ cells (Fig. 1a, c). This is consistent with the fact that, similar to HU, MMS does not cause significant amounts of DSBs at replication forks [36–38]. We also tested UV sensitivity of the above-mentioned mutant cells. UV also stalls replication fork without significantly generating DSBs [39, 40]. NuA4 mutants were not significantly sensitive to UV. Importantly, there was no additive UV sensitivity between *swi1*∆ and *nmt81-vid21*, but UV sensitivity of *swi1*∆ cells was partially rescued by *nmt81-vid21* (Additional file 1: Supplementary Fig. S3A). Therefore, our findings suggest that, unlike the positive role of NuA4 in replication fork repair in response to fork breakage induced by CPT, NuA4 has negative impact on genomic integrity when replication forks are stalled.

We then tested bleomycin sensitivity of NuA4 mutants (Additional file 1: Supplementary Fig. S3B). Bleomycin is a radiomimetic drug that causes single-strand and double-strand DNA breaks without directly interfering with the DNA replication process [41]. Consistent with the role of NuA4 and H4 acetylation in DSB repair, *nmt81-vid21* and *mst1-L344S* cells were sensitive to bleomycin. However, there was no additive bleomycin sensitivity between *nmt81-vid21* and *swi1*∆ (Additional file 1: Supplementary Fig. S3B). Thus, we concluded that the additive genetic interaction we observed between NuA4 mutations and *swi1*∆ is specific to fork breakage during DNA replication. Therefore, hereafter, this study focuses on the role of NuA4 and H4 acetylation in replication fork repair and maintenance in response to CPT.

### NuA4 is required for replication fork recovery after DNA damage

**Fig. 2:**
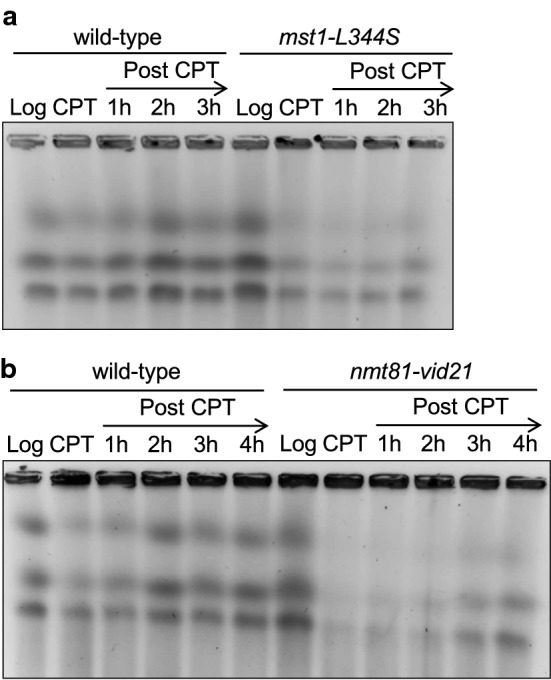
NuA4 is required for replication recovery after DNA damage provoked by CPT. **a**, **b** NuA4 subunits promote efficient recovery of DNA replication after DNA damage. Chromosome samples from the indicated cells were examined by PFGE. Exponentially growing cells (Log) were treated with CPT for 3 h and returned to fresh medium (post-CPT) at 25 °C (in **a**) or 30 °C (in **b**). Cells were collected, and chromosome samples were prepared at the indicated times. Representative images of repeat experiments are shown

We previously reported that *swi1*∆ cells have defects in replication fork recovery after 5 µM CPT treatment [42]. To determine whether NuA4 is required for replication fork recovery after fork breakage, chromosomal DNA from wild-type and *mst1-L344S* cells were prepared before and at 3 h after 15 µM CPT treatment, and also at different time points after the removal of CPT. This concentration was used because *mst1-L344S* cells are only mildly sensitive to CPT at a permissive temperature 25 °C. Chromosomal DNA was then resolved by pulsed-field gel electrophoresis (PFGE), which allows only fully replicated chromosomes to enter the gel (Fig. 2a). Chromosomes prepared from exponentially growing wild-type and *mst1-L344S* cells (log) migrated into the gel. CPT treatment caused replication fork breakage, leading to a reduction in the amounts of chromosomes that migrated into the gel in both wild-type and *mst1-L344S* cells (Fig. 2a). When cells were returned to fresh medium without CPT, chromosomes from wild-type cells were recovered at 1 h after CPT removal due to the completion of DNA synthesis. However, chromosomes from *mst1-L344S* cells did not migrate at the 1 h time point and displayed a reduced capacity to enter the gel at 2 and 3 h during recovery (Fig. 2a). We have also obtained similar defects in replication recovery when *nmt81-vid21* cells were treated with CPT and returned into fresh medium (Fig. 2b). Thus, NuA4 subunits Mst1 and Vid21 are required for recovery of collapsed replication forks after CPT-dependent replication fork breakage.

**Fig. 3:**
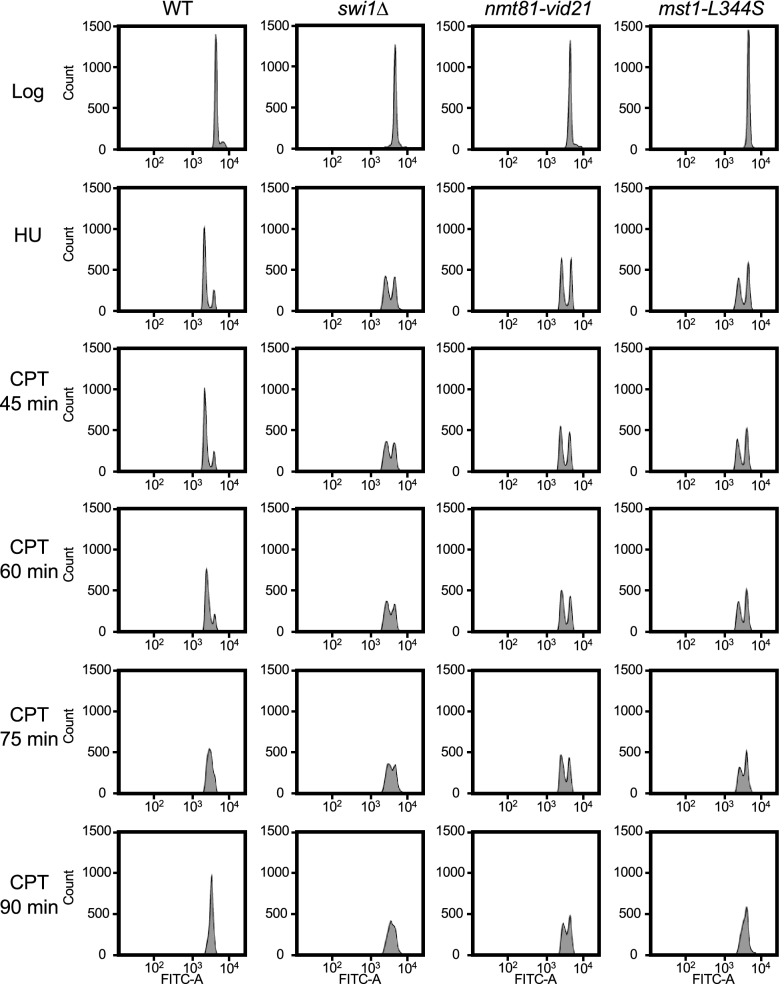
Flow cytometry analysis of NuA4 mutants. The exponentially growing (Log) cells of the indicated genotypes were incubated with 15 mM HU for 5 h at 25 °C to stall cell cycle progression at the beginning of S-phase (HU). Cells were then released into YES medium supplemented with 15 µM CPT for the indicated time and subjected to cell cycle analysis using a FACS LSRFortessa cell analyzer

To further investigate the role of NuA4 in replication fork recovery, we performed flow cytometry analysis of NuA4 mutants. WT, *swi1*∆, *nmt81-vid21*, and *mst1-L344S* cells were incubated in the presence of HU for 5 h at 25 °C in order to arrest cells at the beginning of S-phase with a G1-DNA content. We used 25 °C because *mst1-L344S* cells are temperature-sensitive. Although most wild-type cells were arrested at the onset of S-phase, with unknown reasons, only approximately half or less of *swi1*∆, *nmt81-vid21*, and *mst1-L344S* cells were arrested (Fig. 3). Cells were then released into CPT-containing medium. As shown in Fig. 3, WT cells were able to complete DNA synthesis within 90 min. However, a large portion of *nmt81-vid21* cells still showed a G1-DNA content in 90 min. Although the effects were mild, *swi1*∆ and *mst1-L344S* cells also showed a delay in S-phase progression. Thus, our results strengthen the notion that NuA4 is involved in replication fork recovery in the presence of CPT.

### Histone H4 acetylation is involved in replication fork repair

**Fig. 4:**
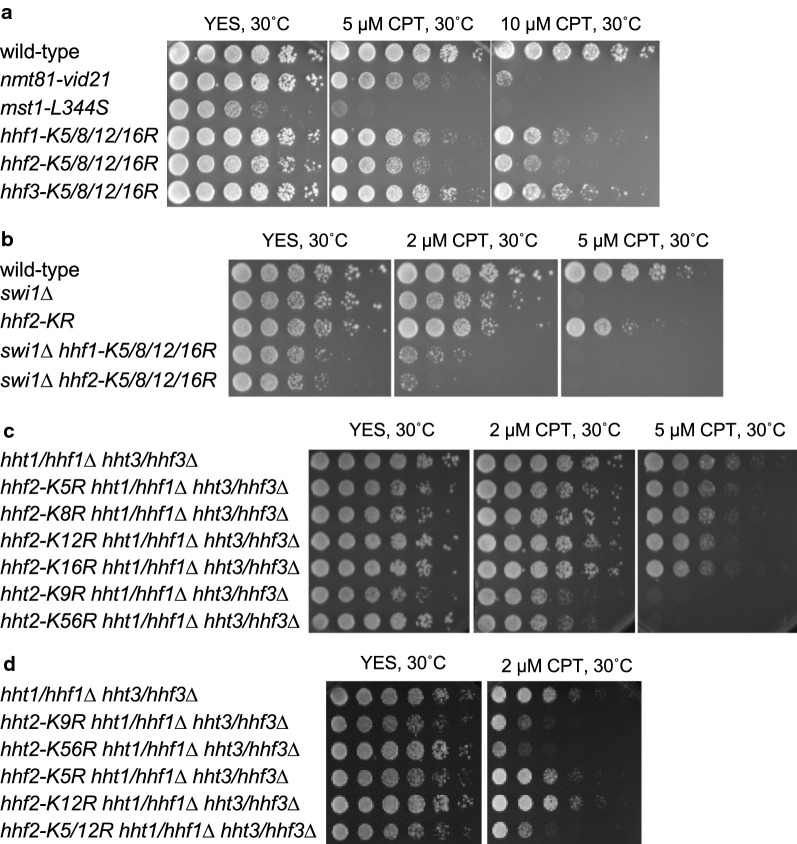
Histone H4 acetylation is require for cellular tolerance to CPT. Fivefold serial dilutions of the cells with the indicated genotypes were incubated on YES agar medium supplemented with the indicated concentration of CPT for 3 to 4 days at 30 °C, and photographed. Representative images of repeat experiments are shown

NuA4 acetylates lysine residues at positions 5, 8, 12 and 16 (K5, K8, K12 and K16) at the N-terminal tail domain of nucleosomal histone H4 [43–46]. Therefore, these residues of each H4 were simultaneously mutated to arginine, mimicking the unacetylated state, resulting in *hhf1-K5/8/12/16R*, *hhf2-K5/8/12/16R*, and *hhf3-K5/8/12/16R* mutants. When we introduced the K5/8/12/16R mutations in all three H4 genes simultaneously, *S. pombe* cells were inviable, indicating that acetylation of H4 tail is essential for cell growth. Therefore, we tested individual H4 mutants (*hhf1-K5/8/12/16R*, *hhf2-K5/8/12/16R* and *hhf3-K5/8/12/16R*) for sensitivity to CPT. We found that all these mutants were sensitive to CPT (Fig. 4a). We also found that *hhf2-K5/8/12/16R* cells were sensitive to MMS, UV, and bleomycin (Fig. 1a, b, Additional file 1: Supplementary Fig. S3B, and S3C).

We then investigated genetic interaction between histone mutations and *swi1*∆ in CPT sensitivity. *swi1*∆ *hhf2-K5/8/12/16R* cells were more sensitive to CPT than *swi1*∆ or *hhf2-K5/8/12/16R* cells (Fig. 4b). These data suggest that H4 acetylation is involved in replication fork repair after fork breakage. In addition, *swi1*∆ *hhf2-K5/8/12/16R* cells grew slower than either single mutant even in the absence of CPT (Fig. 4b), suggesting the role of histone H4 acetylation in protecting replication fork during unperturbed DNA replication. We then generated histone mutants with single amino acid changes. For this purpose, we first generated *hht1/hhf1*∆ *hht3/hhf3*∆ double deletion cells. This genetic background allows us to evaluate the effect of H4 (or H3) mutation by only mutating the *hhf2* (or *hht2*) gene. Accordingly, we constructed strains harboring mutations at K5, K8, K12, and K16 in the *hhf2* gene. These lysine residues were changed to arginine, mimicking the unacetylated state. As a control, we also introduced a mutation at K9 and K56 in *hht2* because these sites are acetylated in response to DNA damage response [47]. Consistent with the proposed roles of H3K9 and H3K56 in DNA damage response, *hht2-K9R* and *hht2-K56R* cells were sensitive to CPT (Fig. 4c). In contrast, *hhf2-K5R*, *hhf2-K8R*, *hhf2-K12R*, and *hhf2-K16R* cells were not significantly sensitive to CPT when compared to control cells (*hht1/hhf1*∆ *hht3/hhf3*∆) (Fig. 4c). Therefore, we introduced multiple mutations in *hhf2* in the *hht1/hhf1*∆ *hht3/hhf3*∆ background. Our investigation revealed that although *hhf2-K5/12/R* cells were viable, the K5/8/12R and K5/8/12/16R mutations were lethal in this background, indicating the importance of multiple acetylation events in supporting *S. pombe* growth. Interestingly, *hhf2-K5/12/R* cells were as sensitive to CPT as *hht2-K9R* and *hht2-K56R* cells (Fig. 4d), indicating the importance of histone H4K5 and H4K12 acetylation in DNA damage response.

**Fig. 5:**
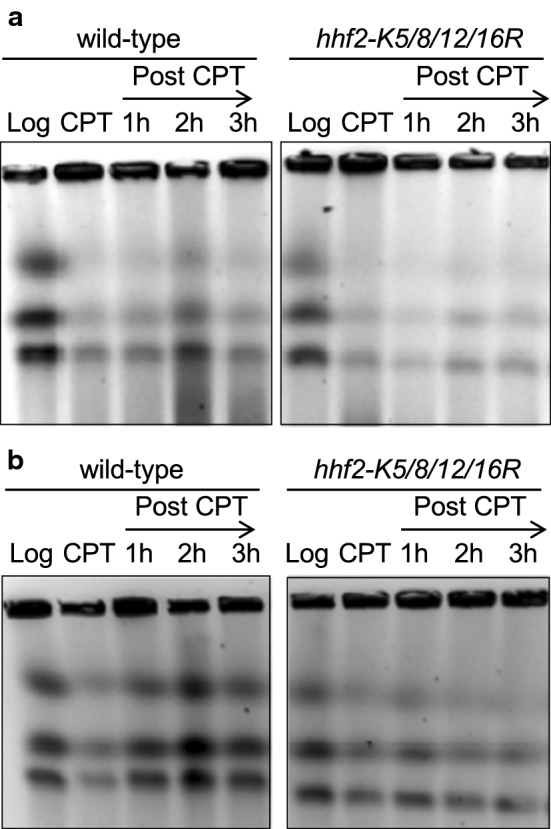
Histone H4 acetylation are required for replication recovery after DNA damage provoked by CPT. **a**, **b** PFGE analysis was performed as described in Fig. 2. histone mutants fail to efficiently recover DNA replication

**Fig. 6:**
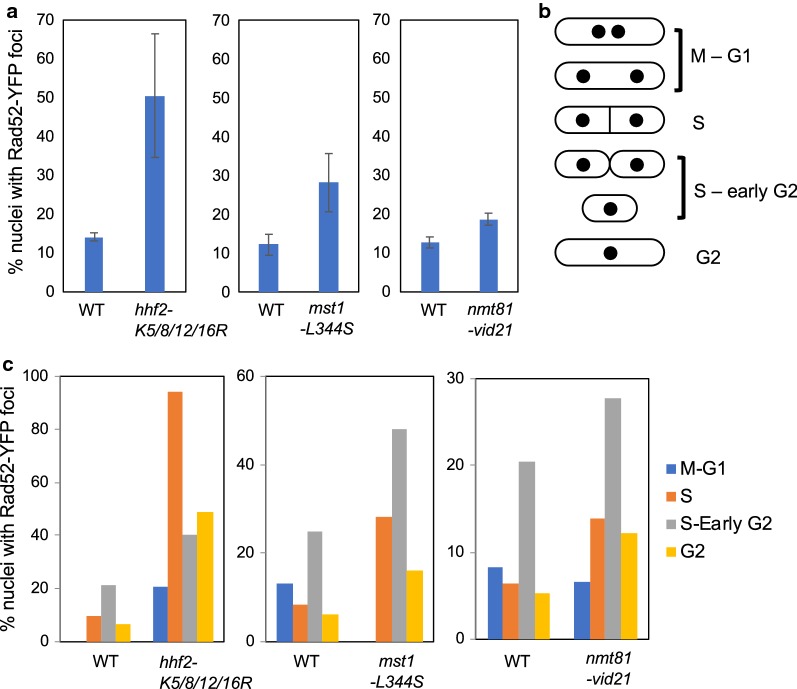
Defects in NuA4 or histone H4 acetylation results in accumulation of DNA damage. **a** Cells of the indicated genotypes were engineered to express Rad52-YFP and grown to mid-log phase at 25 °C. The percentages of nuclei with at least one Rad52-YFP focus are shown. At least 200 cells were counted for each strain in each experiment. Error bars correspond to standard errors of mean (SEM) obtained from three independent experiments. **b** Schematic drawing of nuclear and morphological changes during the *S. pombe* cell cycle. **c** Quantification of Rad52-YFP foci according to cell cycle stages was also conducted using the results obtained in **a**. For this purpose, cell length, nuclei number and position, and the presence of a division plate were analyzed as described in our previous studies [29, 32, 49, 54, 66, 72]

To test the role of histone H4 acetylation in replication fork repair, we examined the replication recovery after fork collapse induced by CPT exposure. As described above, chromosomal DNA from wild-type and H4 mutants were prepared before and at 3 h after CPT treatment, and also at different time points after the removal of CPT. *hhf2-K5/8/12/16R* and *hhf3-K5/8/12/16R* mutants had defects in recovery of replication after CPT treatment (Fig. 5a, b). To further investigate the role of histone H4 during DNA replication, we monitored the localization of Rad52, which is known to form DNA repair foci at the site of DNA damage. *hhf2-K5/8/12/16R* cells were engineered to express Rad52-YFP from its genomic locus. There was a dramatic increase in Rad52 foci formation in *hhf2-K5/8/12/16R* cells (Fig. 6a). We also found a significantly elevated level of Rad52 foci formation in *mst1-L344S* cells (Fig. 6a), which is consistent with a previous study [25]. Furthermore, *nmt81-vid21* cells also had an increase in Rad52 foci formation (Fig. 6a). To determine whether the DNA damage arose from replication abnormalities, cell cycle position of cells containing Rad52-YFP foci was evaluated. Cell cycle positions in fission yeast can be estimated by noting cell length, nuclear morphology, and the appearance of septum as shown in Fig. 6b. This analysis demonstrated that Rad52-YFP foci occurred predominantly in S or early G2 phase (Fig. 6c), suggesting that replication fork abnormality causes spontaneous DNA damage in *hhf2-K5/8/12/16R* and NuA4 mutants.

**Fig. 7:**
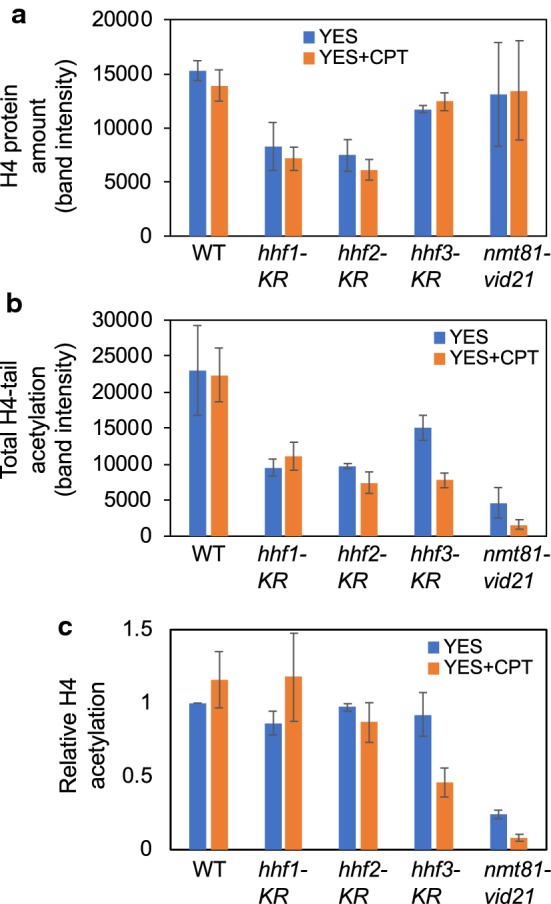
Levels of histone H4 and histone H4 acetylation are altered in histone H4 mutants. Cells of the indicated genotypes were grown to mid-log phase and incubated in the absence or presence of 15 µM CPT. Western blotting of whole-cell extracts was performed using the anti-H4 and anti-acetyl (K5/8/12/16) histone H4 antibody. **a**, **b** Quantification of H4 protein amounts (in **a**) and total levels of H4 acetylation (in **b**) was performed using Image J software. **c** The level of H4 acetylation was also normalized to the total H4 level in each cell extract. Error bars correspond to standard errors of mean (SEM) obtained from three independent experiments

**Fig. 8:**
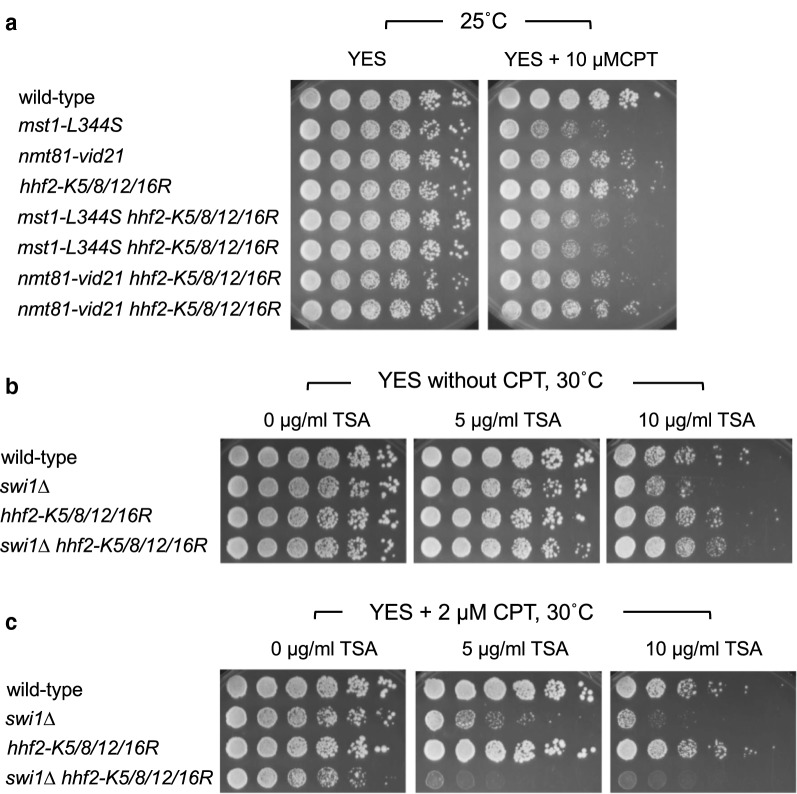
NuA4 functions through H4 acetylation for cellular tolerance to CPT. Fivefold serial dilutions of the cells with the indicated genotypes were incubated on YES agar medium supplemented with the indicated concentration of CPT and/or TSA for 3–4 days at 30 °C, and photographed

We previously showed that mutations in NuA4 components (*nmt81-vid21* and *mst1-L344S*) caused a reduction in the levels of H4-tail acetylation [24]. We also monitored H4 and H4-tail acetylation levels in H4 mutant cells (*hhf1-K5/8/12/16R*, *hhf2-K5/8/12/16R*, and *hhf3-K5/8/12/16R*). Interestingly, the H4 protein amounts were generally reduced in these cells with or without CPT treatment (Fig. 7a). As a result, the total levels of acetylated H4-tail were also considerably reduced in these H4 mutant cells (Fig. 7b), although there was no significant difference in H4 acetylation when normalized to the amounts of the H4 protein in each mutant, except for CPT-treated *hhf3-K5/8/12/16R* cells (Fig. 7c). The total H4-tail acetylation and normalized H4 acetylation levels were both dramatically reduced in *nmt81-vid21* cells (Fig. 7b, c). These findings prompted us to investigate whether NuA4 protects replication fork through H4 acetylation, we performed epistasis analysis between NuA4 and H4 mutations in the cellular tolerance to CPT. Accordingly, CPT sensitivity of *nmt81-vid21* *hhf2-K5/8/12/16R* and *mst1-L344S* *hhf2-K5/8/12/16R* cells were compared to corresponding single mutants. There was no significant additive effect on CPT sensitivity between NuA4 and H4 (Fig. 8a), suggesting that NuA4 functions through H4 acetylation for cellular tolerance to CPT.

A previous study reported that mutations that mimic acetylated state of H4 rescued CPT sensitivity of *tof1*∆ cells (Tof1 is the *S. cerevisiae* ortholog of Swi1) [48]. Therefore, to further understand the role of histone H4 acetylation in the regulation of replication fork repair, we treated *swi1* and H4 mutant cells with CPT and the histone deacetylase (HDAC) inhibitor, trichostatin A (TSA), which pharmacologically increases acetylated H4. As shown in Fig. 8b, *swi1*∆ cells were sensitive to TSA (Fig. 8b). Interestingly, unlike the case for *S. cerevisiae* [48], CPT sensitivity of *swi1*∆ cells was further enhanced by an increase in acetylated H4 via TSA treatment (Fig. 8c). TSA also led to further increase in CPT sensitivity of *swi1*∆ *hhf2-K5/8/12/16R* cells. Considering that *hhf2-K5/8/12/16R* mutation and TSA have opposite effects on histone H4 acetylation, our results suggest the importance of fine-tuning histone acetylation in replication fork repair in response to CPT. Interestingly, when cells were not treated with CPT, the TSA sensitivity of *swi1*∆ was suppressed by *hhf2-K5/8/12/16R* mutation (Fig. 8c), suggesting hyperacetylation caused by TSA plays a negative role in the absence of Swi1 and that *hhf2-K5/8/12/16R* mutation reversed this negative effect. The results also suggest that histone acetylation has negative impact on unperturbed DNA replication when Swi1 is defective.

### The Swi1–Swi3 replication fork protection complex interacts with the NuA4 subunit Vid21

**Fig. 9:**
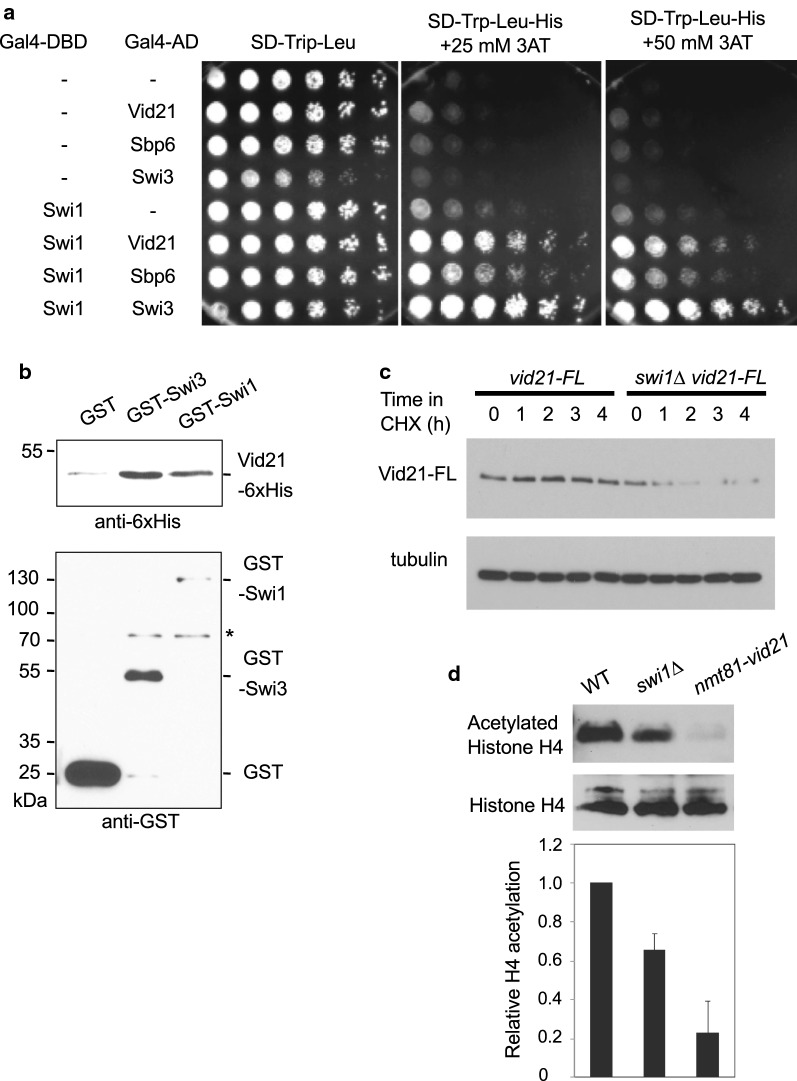
Swi1 interacts with Vid21, a regulatory subunit or the NuA4 complex, and is involved in efficient acetylation of histone H4. **a** Two-hybrid interaction between Swi1 and Vid21. Swi1 fused to Gal4-DBD (DNA-binding domain) was tested in the Y190 reporter strain for interaction when combined with Vid21 (C-terminal regions: amino acids 593-985) fused to Gal4-AD (activation domain). Y190 cells harboring Swi1-DBD and Vid21-AD were able to grow in the presence of 3-aminotrizole (3AT). Swi3-AD and Sbp6-AD (Swi1-binding protein 6) were used as positive controls that interact with Swi1. **b** The indicated GST-fusion proteins were expressed in and purified from *S. pombe* cells. Glutathione Sepharose beads bound to the indicated GST-fusion proteins were incubated with recombinant Vid21_593-985_-6xHis. The beads were washed and analyzed by Western blotting using the anti-6xHis antibody. Asterisk shows non-specific bands. **c** Exponentially growing cells of the indicated genotypes were treated with 100 µg/ml of CHX for the indicated times. Whole-cell extracts were prepared at the indicated times and probed with the anti-FLAG M2 antibody. Western blotting of tubulin was performed as a loading control. Representative images of repeat experiments are shown. **d** Western blotting of whole-cell extracts was performed using the anti-H4 and anti-acetyl (K5/8/12/16) histone H4 antibody. Quantification of H4 acetylation was performed using Image J software. The level of H4 acetylation was normalized to the total H4 level in each cell extract. Error bars correspond to standard errors of mean (SEM) obtained from three independent experiments

Swi1 is required for recovery of collapsed replication forks in *S. pombe* [32, 49]. To further understand the mechanism of DNA replication fork maintenance, we previously employed the yeast two-hybrid system to screen an *S. pombe* cDNA library for identification of Swi1-interacting proteins [50]. Among 1 × 10^6^ *S. cerevisiae* colonies transformed with an *S. pombe* cDNA library, 104 formed colonies in the presence of 25 mM 3-aminotriazole (3-AT). After DNA sequencing analysis, 11 clones were found to contain a cDNA encoding the amino acids 593-985 region of the *S. pombe* homolog of Vid21/Eaf1 (Vid21_593-985_ hereafter). As shown in Fig. 9a, *S. cerevisiae* reporter cells expressing Swi1 fused to Gal4 DNA-binding domain (Gal4-DBD) and Vid21_593-985_ fused to Gal4 activation domain (Gal4-AD) showed a robust growth in the presence of 3-AT, indicating that Swi1 interacts with Vid21 in a two-hybrid assay. To establish the physical association between Swi1 and Vid21, we first performed immunoprecipitation of Vid21 and Swi1 expressed as epitope-tagged proteins in *S. pombe* cells. However, we were not able to detect interaction between Vid21 and Swi1 with this method. It is possible that the Vid21-Swi1 interaction is weak or transient. Therefore, GST-Swi1 expressed in and purified from *S. pombe* cells was incubated with recombinant His_6_-Vid21_593-985_. We also purified GST-Swi3 from *S. pombe* cells and incubated with His_6_-Vid21_593-985_. As shown in Fig. 9b, His_6_-Vid21_593-985_ coprecipitated with GST-Swi1 and GST-Swi3, indicating that Vid21 interacts with the Swi1–Swi3 fork protection complex.

### Swi1 mediates Vid21 stabilization and proper histone H4 acetylation

The physical interaction between Swi1 and Vid21 prompted us to investigate a role of Swi1 in the regulation of NuA4. For this purpose, we first investigated the stability of Vid21 in the absence of Swi1 (Fig. 9c). Accordingly, cells expressing FLAG-tagged Vid21 (Vid21-FL) from its endogenous promoter were treated with cycloheximide (CHX), a compound that inhibits new protein synthesis, allowing for the determination of protein stability. Vid21-FL was found to be stable in the wild-type background. In sharp contrast, Vid21-FL showed a dramatic instability in *swi1*∆ cells, indicating that Vid21 is unstable in the absence of Swi1 (Fig. 9c). Because Vid21 is a subunit of NuA4 histone H4 acetyltransferase, we examined the levels of histone H4 acetylation in *swi1*∆ cells. As shown in Fig. 9d, *swi1*∆ cells displayed inefficient histone H4 acetylation when compared to wild-type cells (Fig. 9d). *nmt81-vid21* cells also showed defects in histone H4 acetylation as previously described (Fig. 9b). Therefore, our results are consistent with the notion that Swi1 mediates Vid21 stabilization and promotes histone H4 acetylation. Our results also suggest that Swi1’s function in replication fork recovery may be partly attributed to Swi1’s role in Vid21 stabilization, which may contribute to NuA4 activity.

## Discussion

The replication machinery encounters a variety of obstacles on the chromosome, including damaged DNA templates. In addition, a number of chromosomal regions are considered to be difficult to replicate owing to secondary DNA structures and DNA-binding proteins. Indeed, highly compact chromatin structures present significant barriers to fork progression [51, 52]. Therefore, chromatin state, which is regulated by histone modifications, plays an important role in proper progression of the replication fork.

We previously demonstrated that two double bromodomain-containing proteins, Bdf1 and Bdf2, are involved in recovery of DNA replication after DNA damage caused by CPT, a topoisomerase poison that causes replication fork breakage [24]. Bromodomain-containing proteins are known to recognize and stabilize acetylated histone H4 [53]. Indeed, the level of acetylated histone H4 was reduced in both *bdf1*∆ and *bdf2*∆ cells in fission yeast [24]. These findings indicate the role of histone H4 acetylation in replication recovery after DNA damage. Consistently, in this present study, we demonstrated that histone H4 acetylation at NuA4-targeted sites plays an important role in recovery of DNA replication after DNA damage.

Unlike H3K9 or H3K56 mutations, a single acetylation-site mutation in H4 failed to sensitize *S. pombe* cells to a DNA-damaging agent (Fig. 4c). However, H4K5/12R mutant cells showed sensitivity to CPT (Fig. 4d). Furthermore, although the K5/8/12/16/R mutation is lethal to *S. pombe* when introduced to all three histone H4 genes, cells with the *K5/8/12/16/R* mutation in a single H4 gene were viable and displayed sensitivity to CPT (Fig. 4a). Therefore, it is highly likely that all 4 NuA4-dependent acetylation sites contribute to replication fork repair.

**Fig. 10:**
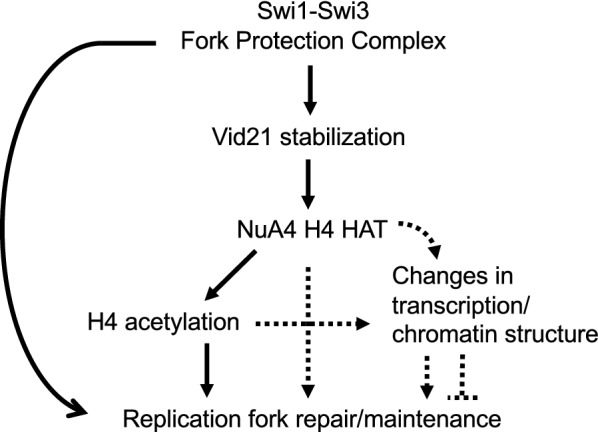
Model of Swi1–Swi3-mediated regulation of NuA4 in replication fork repair and maintenance

NuA4 has been shown to be involved in genome maintenance. Deficiency in NuA4 subunits sensitizes cells to various DNA-damaging agents. Although the roles of NuA4 in DNA repair are relatively well characterized [2], its role in DNA replication is still elusive. In this regard, we showed that *S. pombe* Swi1 interacts with Vid21, a regulatory subunit of the NuA4 histone acetyltransferase complex (Fig. 9). Strikingly, Vid21 showed a dramatic instability in the absence of Swi1 (Fig. 9c). We previously showed that several replisome components including DNA polymerases and helicases undergo degradation in the absence of Swi1 [42]. Because Swi1 prevents fork collapse, the degradation of Vid21 could be indirect consequence of replication stress elevated in the absence of Swi1. However, considering that Swi1–Swi3 interacts with Vid21, it is straightforward to suggest that Swi1 directly prevents degradation of Vid21 to promote efficient recruitment or stable association of NuA4 at the replication fork. Consistently, loss of Swi1 leads to a significant reduction in the level of histone H4 acetylation (Fig. 9d). Therefore, we propose that Swi1 promotes NuA4-mediated histone H4 acetylation at the replication fork to facilitate DNA repair (Fig. 10).

In this report, we showed additive genetic interaction between NuA4 mutations and *swi1*∆ in CPT sensitivity. In contrast, NuA4 mutations partially suppressed *swi1*∆ cells’ sensitivity to fork-stalling agents including HU, MMS and UV (Fig. 1, Additional file 1: Supplementary Fig. S3) [24]. It is suggested that highly transcribed genes are prone to DNA damage when replication forks are unstable [51]. Considering that histone acetylation generally upregulates transcription, it is possible that histone acetylation can induce DNA damage probably by increasing transcription, thereby elevating the chances of collisions between replication and transcription. In the absence of Swi1, where cells experience replication stress, mutations in NuA4 appear to prevent genomic instability probably by reducing the risk of collisions between the transcription and replication forks. In this scenario, NuA4-mediated H4 acetylation has negative impact on genomic instability during fork stalling. However, in the presence of CPT, where cells experience DSBs at the replication forks especially in *swi1*∆ cells, NuA4 and histone H4 acetylation appear to play positive role in preventing and/or repairing DSBs during DNA replication (Fig. 10).

Interestingly, Swi1 contains the DDT domain [54], which is found in various chromatin-remodeling factors [55]. Furthermore, Swi1 is required for suppression of DNA damage at difficult-to-replicate chromosome regions including heterochromatin and highly transcribed genes [56–58]. Therefore, it is highly possible that Swi1 functions as a fork-associated chromatin-regulating factor to ensure timely acetylation of histone H4 and promote proper progression and repair of replication fork. Although we prefer the idea that NuA4-mediated H4 acetylation directly facilitates replication fork repair, CPT sensitivity of *mst1-L344S* cells was not completely recovered to wild-type level. Therefore, there seems to be a minor contribution where NuA4 indirectly modulates DNA repair processes via changes in gene expression or modulation of other target proteins such as H2A and H2AZ. Such changes or modulations may also affect local chromatic structures to modulate DNA repair processes (Fig. 10).

Studies have shown that Swi1 and its orthologs play critical roles in replication fork protection in response to DNA-damaging agents [31]. In addition, Swi1 stabilizes the replisome at natural impediments to the DNA replication process [51]. Such impediments are found at various sites of the chromosomes and include the *S. pombe* mating-type locus, rDNA-pausing sites, highly transcribed chromosomal regions, and telomeres. These regions are difficult to replicate and can cause genomic instability if not properly regulated [51]. Interestingly, many of these difficult-to-replicate sites contain the Myb/SANT family of DNA-binding proteins, which are recruited to specific chromosome loci. For example, the Myb/SANT protein Rtf1 binds to the RTS1 site of the mating-type locus in fission yeast. Importantly, Rtf1 functions to terminate replication fork movement at this locus in a Swi1-dependent manner [59]. Reb1, another Myb/SANT domain protein, is recruited to rDNA-pausing sites and mediates Swi1-dependent replication fork pausing [60, 61]. Furthermore, we recently showed that Swi1 and its human ortholog Timeless interact with telomere-binding proteins Tbf1 and TRF1, respectively [62, 63]. Tbf1 and TRF1 also harbor the Myb/SANT domain. Accordingly, we hypothesized that Swi1 interacts with Myb/SANT domain proteins to protect replication forks at difficult-to-replicate sites [62, 63]. Intriguingly, Vid21 also has the Myb/SANT domain [34]. Considering that highly transcribed regions require the function of NuA4-mediated histone acetylation, and such regions are difficult to replicate, it is highly possible that Swi1–Vid21 interaction mediates proper DNA replication process at highly transcribed regions that require chromatin remodeling and/or organization. Further investigation warrants a better understanding of the role of Swi1-mediated chromatin regulation in coordinating DNA replication and repair processes at the replication fork.

## Conclusions

Based on our findings described in this study, we conclude that NuA4 acetyltransferase and histone H4 acetylation play critical roles in repair of broken replication forks. Although further investigation is needed to precisely understand the role of Swi1–Vid21 interaction in DNA replication process, our results suggest that Swi1 facilitates NuA4 function to promote histone H4 acetylation at the replication fork in order to repair replication forks upon replication stress. Considering that structural changes of chromatin and transcriptional activities are regulated by epigenetic modifications of histone proteins, elucidating how histone modifications are coordinated with DNA replication and repair is of high significance. Therefore, this study, which links replication fork protection with histone H4 acetylation, should open new directions to further understand the role of histone regulation in replication and repair program.

## Methods

### General techniques

*Schizosaccharomyces pombe* strains were grown in Yeast Extract + Supplements (YES) media (0.5% yeast extract, 3% glucose) supplemented with the appropriate amino acids. The methods used for genetic and biochemical analyses of fission yeast have been previously described [64, 65]. Western blotting and drug sensitivity assays were performed as described in our earlier studies [42, 66]. Microscopic analyses of yellow fluorescent protein (YFP) were performed using Olympus PROVIS AX70 microscope equipped with a Retiga EXi camera (QImaging, Surrey, BC, Canada). Images were acquired with iVision software (BioVision Technologies, Exton, PA) and analyzed with ImageJ software (National Institutes of Health, Bethesda MD). For Rad52-YFP analyses, approximately 200 cells were counted for each experiment, as described previously [66].

### Plasmids and site-directed mutagenesis

*Schizosaccharomyces pombe* possesses three gene pairs encoding histone H3 (Hht) and H4 (Hhf): *hht1/hhf1*, *hht2/hhf2*, and *hht3/hhf3* [67]. These histone H3-H4 gene pairs were amplified by PCR from the genomic DNA preparation and cloned into the pBluescript II SK (+) vector containing hygromycin (hphMX6), nourseothricin (natMX6), and kanamycin (kanMX6)-resistant genes, respectively, to generate pBluescript II-hht1-hhf1-hphMX6, pBluescript II-hht2-hhf2-natMX6, and pBluescript II-hht3-hhf3-kanMX6. The K5/8/12/16R (KR, hereafter) mutation in histone H4 (hhf) genes was introduced by Kunkel site-directed mutagenesis in these plasmids, resulting in pBluescript II-hht1-hhf1-KR-hphMX6, pBluescript II-hht2-hhf2-KR-natMX6, and pBluescript II-hht3-hhf3-KR-kanMX6. The K5R, K8R, K12R, K16R, and K5/12R mutations in histone H4.2 (*hhf2*) gene were introduced into pBluescript II-hht2-hhf2-natMX6, to generate pBluescript II-hht2-hhf2-K5R-kanMX6, pBluescript II-hht2-hhf2-K8R-kanMX6, pBluescript II-hht2-hhf2-K12R-kanMX6, pBluescript II-hht2-hhf2-K16R-kanMX6, and pBluescript II-hht2-hhf2-K5/12R-kanMX6. K9R and K56 mutations in histone H3.2 (*hht2*) genes were also introduced into pBluescript II-hht2-hhf2-natMX6, to generate pBluescript II-hht2-K9R-hhf2-kanMX6 and pBluescript II-hht2-K56R-hhf2-kanMX6. The mutated histone gene pair fragments containing appropriate drug markers excised from these vectors were used to generate histone mutants described in the next section.

To express N-terminal GST-fused Swi1 in *S. pombe*, the full-length *swi1* open-reading frame was amplified by PCR and fused to GST at the NdeI/NcoI site in pREP-KZ [68], resulting in pREP-KZ-Swi1. The full-length *swi3* open-reading frame was also amplified by PCR and inserted at the NdeI/NotI site of pREP-KZ to construct pREP-KZ-Swi3. For bacterial expression of hexahistidine-tagged Vid21, the amino acid region 593-985 of the vid21 open-reading frame was amplified by PCR and inserted into the EcoRI/XhoI site of pET28a (Novagen), resulting in pET28a-Vid21CT. To express Rad52-YFP in *S. pombe* cells, the 1.5 kb NotI-BglII fragment containing a C-terminal region of the rad52 open-reading frame fused to YFP cDNA (Rad52CT-YFP) [49, 69] was introduced into NotI/BamHI site of pJK148, resulting in pJK148-Rad52CT-YFP.

In yeast two-hybrid assays, pGAD424 (Clontech) and pAS404 vectors [70] were used to express proteins fused to Gal4 activation and Gal4 DNA-binding domains, respectively.

### *Schizosaccharomyces pombe* strains

The *S. pombe* strains used in this study were constructed using standard techniques and their genotypes are listed in Additional file 2: Supplementary Table S1.

*vid21-5FLAG* (*vid21-5FLAG*::*kanMX6*) was generated by a two-step PCR method [71] to construct a 5FLAG tag at the C terminus of *vid21*. To visualize Rad52-YFP in various mutants, pJK148-Rad52CT-YFP was linearized by AflII and integrated at the *rad52* locus of the mutants.

*hhf1-KR*, *hhf2-KR*, and *hhf3-KR* mutants (K5/8/12/16R mutants): Histone H3-H4 gene pairs *hht1/hhf1*, *hht2/hhf2*, and *hht3/hhf3* were individually deleted using the two-step PCR method to generate *hht1/hhf1*::*natMX6* (*hht1/hhf1*∆), *hht2/hhf2*::*kanMX6* (*hht2/hhf2*∆), and *hht3/hhf3*::*hphMX6* (*hht3/hhf3*∆). To generate the *hhf1-KR*, *hhf2-KR*, and *hhf3-KR* mutants, the *hht1/hhf1*::*natMX6*, *hht2/hhf2*::*kanMX6*, and *hht3/hhf3*::*hphMX6* genes were replaced with *hht1-hhf1-KR-hphMX6*, *hht2-hhf2-KR-natMX6*, and *hht3-hhf3-KR-kanMX6*, respectively. Proper integration of mutated histone H4 genes was confirmed by antibiotic-marker switch and DNA sequencing analysis of histone H4 genes amplified from the mutants. These mutants have mutations in only one of the 3 histone H4 genes and the other two histone H4 genes are intact.

*hhf2-K5R*, *K8R*, *K12R*, *K16R*, *K5/12/R*, *hht2-K9R*, and *K56R* mutants: The *hht2/hhf2*::*kanMX6* gene was replaced with *hht2-hhf2-K5R-kanMX6*, *hht2-hhf2-K8R-kanMX6*, *hht2-hhf2-K12R-kanMX6*, *hht2-hhf2-K16R-kanMX6*, *hht2-hhf2-K5/12R-kanMX6*, *hht2-K9R-hhf2-kanMX6*, and *hht2-K56R-hhf2-kanMX6*. Resulting strains were crossed with an *hht1/hhf1*∆ *hht3/hhf3*∆ strain, to generate *hhf2-K5R*, *K8R*, *K12R*, *K16R*, *K5/12/R*, *hht2-K9R*, and *K56R* mutants. These mutants were confirmed by antibiotic resistance and DNA sequencing analysis of histone H4 or H3 genes amplified from the mutants. These mutants have only one histone gene pair (*hht2-hhf2*), which is mutated.

Mutations and epitope-tagged genes have previously been described for *swi1*∆ [49], *mst1-L344S* [24], and *nmt81-vid21* [24]. *vid21-13Myc* was provided by Dr. Paul Russell. *Saccharomyces cerevisiae* reporter strain Y190 (Clontech) was used for two-hybrid assays.

### Protein purification and in vitro protein interaction assay

Wild-type *S. pombe* cells were transformed with pREP-KZ, pREP-KZ-Swi1 or pREP-KZ-Swi3 and cultured in Edinburgh minimal medium (EMM) without thiamine to overexpress the GST, GST-Swi1, or GST-Swi3 protein. Purification of GST and GST-fused proteins from these cells was performed using Glutathione Sepharose 4B (GS4B, GE Healthcare) as described in our previous study [54]. Purification of His_6_-Vid21CT from E. coli BL21 (DE3) cells that carry pET28a-Vid21C was also performed as described [54].

GS4B beads bound to GST, GST-Swi1, or GST-Swi3 were mixed with His_6_-Vid21CT in buffer A [50 mM Tris–HCl pH 8.0, 150 mM NaCl, 0.1% NP-40, 10% glycerol, 50 mM NaF, 1 mM Na_3_VO_4_, 5 mM EDTA, 5 mM N-methylmaleimide, 1 µM microcystin-LR, 0.1 µM okadaic acid, and Halt protease inhibitor cocktail (Life Technology)], incubated by rotation for 1 h at 4 °C, washed three times with buffer A and once with buffer B (buffer A with 500 mM NaCl) and analyzed by Western blotting using anti GST and hexahistidine antibodies.

### Cell extract preparation, immunoprecipitation, and detection of histone H4 acetylation

To examine protein stability, exponentially growing cells were treated with 100 µg/ml of cycloheximide and collected. Whole-cell extracts were prepared as previously described in our studies [42]. Immunoprecipitation was performed as described [30] using the anti-Myc 9E10 (Covance) with protein G Sepharose, and myc- and FLAG-tagged proteins were detected with the anti-FLAG M2 (Sigma-Aldrich) and anti-Myc 9E10 antibodies, respectively.

For detection of histone H4 acetylation, whole-cell extracts were probed with the anti-acetyl-histone H4 antibody that recognizes histone H4 acetylated at the lysine 5, 8, 12, and 16 residues (06-088, EMD Millipore) as described [24]. Total levels of histone H4 were analyzed by the anti-histone H4 antibody (ab10158, Abcam).

### Pulse-field gel electrophoresis

Exponentially growing cells were treated with 15 µM camptothecin (CPT) for 3 h at 30 °C and then washed and returned to fresh YES medium. Cells were collected at the indicated times, and chromosomal DNA samples were prepared in agarose plug and analyzed with CHEF-DRII system (Bio-Rad) as previously described [66].

### Cell cycle analysis

Exponentially growing cells were treated with 15 mM HU for 5 h at 25 °C to arrest them at the beginning of S-phase. Cells were washed with YES and released into YES medium supplemented with 15 µM CPT. Cells (2 × 10^6^) were collected at the indicated times, fixed in the presence of cold 70% ethanol and washed with cold water. Cells were then treated with 100 µg/ml RNase at 37 °C for overnight, incubated in the presence of 0.5 µM SYTOX Green (ThermoFisher Scientific), sonicated to separate cells and analyzed by a FACS LSRFortessa cell analyzer (BD Biosciences) using FACS Diva software (BD Biosciences). Cell cycle analysis was performed using FlowJo software (Tree Star).

## Additional files

**Additional file 1: Supplementary Figure S1.** Vid21 interacts with Mst1, the catalytic subunit of the NuA4 complex. *S. pombe* cell extracts expressing Mst1-FL and/or Vid21-Myc were subjected to immunoprecipitation using the anti-Myc 9E10 monoclonal antibody and analyzed by Western blotting using the anti-Myc 9E10 or anti-FLAG M2 monoclonal antibody. WCE, whole-cell extract. IP, immunoprecipitation. Asterisks indicate background bands due to cross-reactivity of the antibodies used. **Supplementary Figure S2.** *top1*∆ rescues CPT sensitivity of NuA4 mutants. (A, B, C) Fivefold dilutions of cells with the indicated genotypes were incubated on YES agar medium supplemented with the indicated concentration of CPT or MMS for 3 to 5 days at the indicated temperature. Representative images of repeat experiments are shown. **Supplementary Figure S3.** Genetic interaction between NuA4 mutation and *swi1*∆ in UV and bleomycin sensitivities. (A) Fivefold dilutions of cells with the indicated genotypes were plated on YES agar medium, exposed to the indicated dose of UV, and incubated for 3 days at 30 °C. (B) Fivefold dilutions of cells with the indicated genotypes were incubated on YES agar medium supplemented with the indicated concentration of bleomycin for 3 to 5 days at the indicated temperature. Representative images of repeat experiments are shown.

**Additional file 2: Table S1.** *S. pombe* strains used in this study.

## Abbreviations

3-AT: 3-aminotriazole
CTP: camptothecin
CHX: cycloheximide
DDT: DNA-binding homeobox and different transcription factors
FPC: fork protection complex
GST: glutathione S-transferase
MMS: methyl methanesfulfonate
PFGE: pulsed-field gel electrophoresis
SANT: SWI3, ADA2, N-CoR, and TFIIIB
YES: yeast extract + supplements
YFP: yellow fluorescent protein
