# Supplementary figures and images for: The NuA4 acetyltransferase and histone H4 acetylation promote replication recovery after topoisomerase I-poisoning
Source: Epigenetics Chromatin. 2019 Apr 16;12:24. doi: 10.1186/s13072-019-0271-z (PMC6466672; doi:10.1186/s13072-019-0271-z)

## Supplementary Figure S1

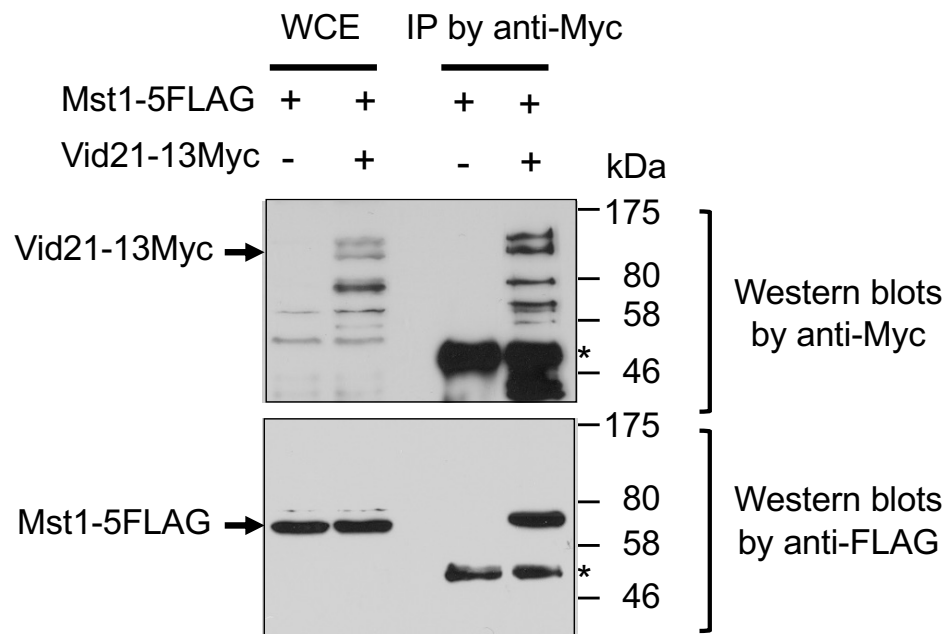

## Supplementary Figure S2

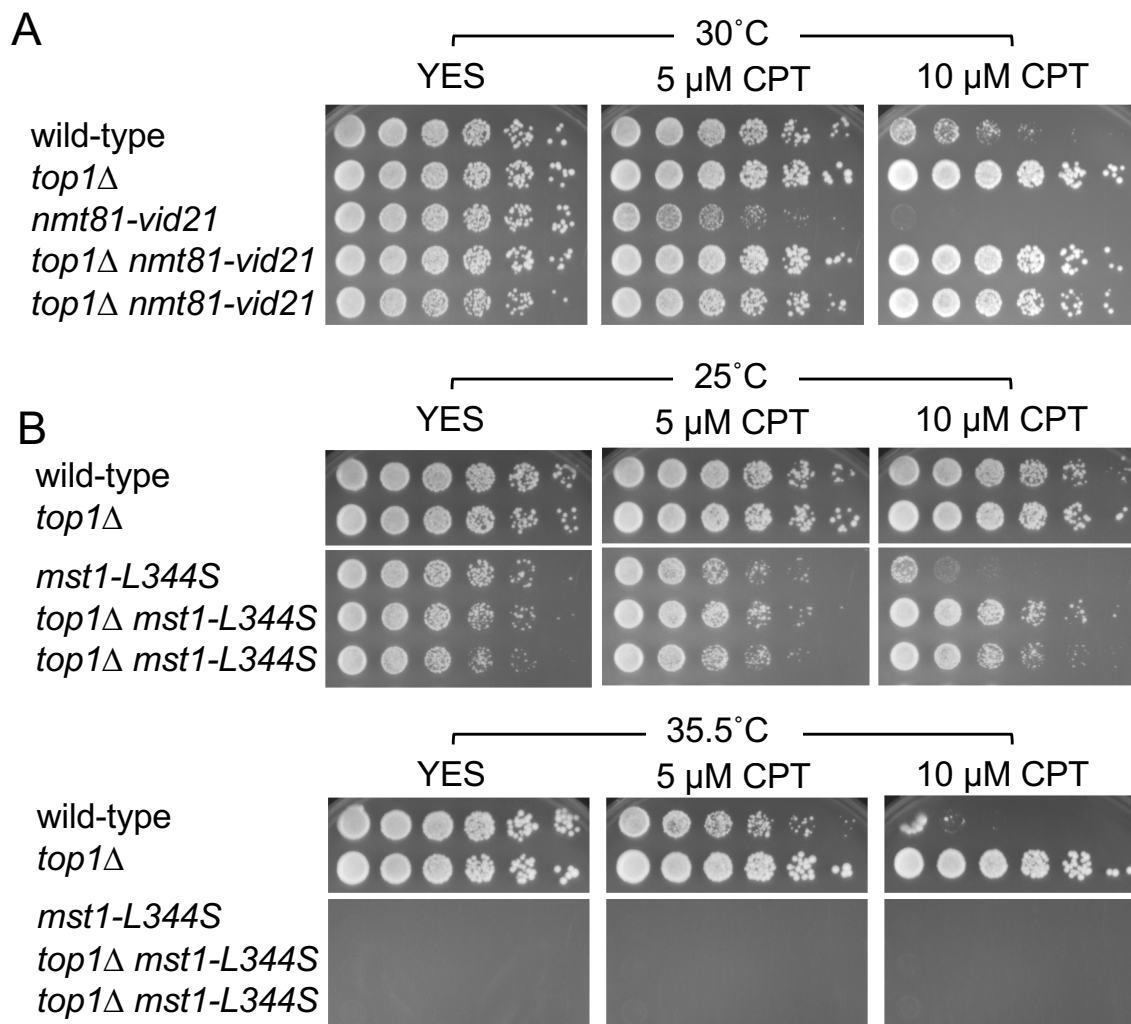

Supplementary Figure S3

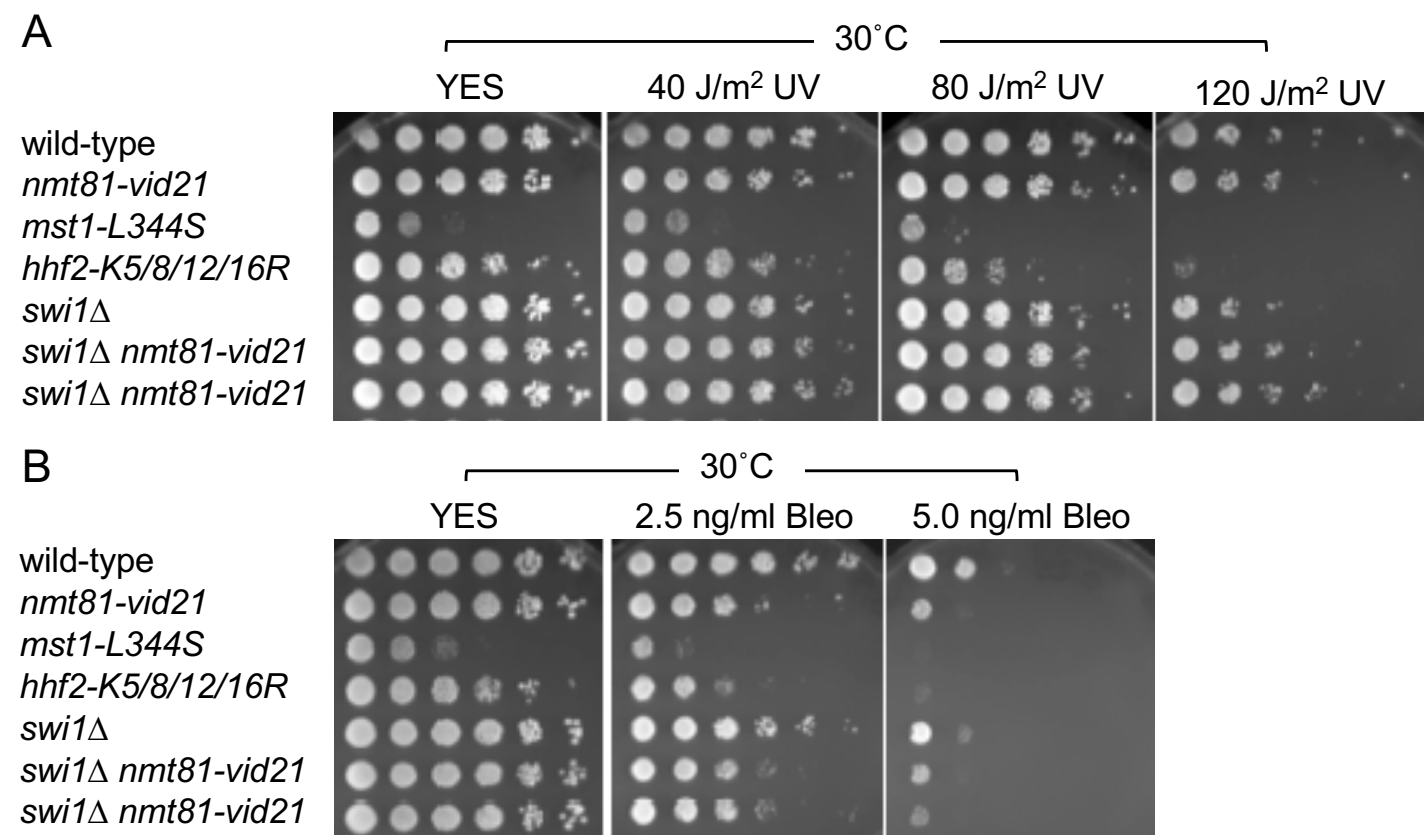

Supplement: Supplementary file 1 — Additional file 1: Supplementary Figure S1. Vid21 interacts with Mst1, the catalytic subunit of the NuA4 complex. S. pombe cell extracts expressing Mst1-FL and/or Vid21-Myc were subjected to immunoprecipitation using the anti-Myc 9E10 monoclonal antibody and analyzed by Western blotting using the anti-Myc 9E10 or anti-FLAG M2 monoclonal antibody. WCE, whole-cell extract. IP, immunoprecipitation. Asterisks indicate background bands due to cross-reactivity of the antibodies used. Supplementary Figure S2. top1∆ rescues CPT sensitivity of NuA4 mutants. (A, B, C) Fivefold dilutions of cells with the indicated genotypes were incubated on YES agar medium supplemented with the indicated concentration of CPT or MMS for 3 to 5 days at the indicated temperature. Representative images of repeat experiments are shown. Supplementary Figure S3. Genetic interaction between NuA4 mutation and swi1∆ in UV and bleomycin sensitivities. (A) Fivefold dilutions of cells with the indicated genotypes were plated on YES agar medium, exposed to the indicated dose of UV, and incubated for 3 days at 30 °C. (B) Fivefold dilutions of cells with the indicated genotypes were incubated on YES agar medium supplemented with the indicated concentration of bleomycin for 3 to 5 days at the indicated temperature. Representative images of repeat experiments are shown. [file 13072_2019_271_MOESM1_ESM.pdf]
